# Targeting the NLRP3 Inflammasome in Severe COVID-19

**DOI:** 10.3389/fimmu.2020.01518

**Published:** 2020-06-23

**Authors:** Tracey L. Freeman, Talia H. Swartz

**Affiliations:** Division of Infectious Diseases, Department of Medicine, Immunology Institute, Icahn School of Medicine at Mount Sinai, New York, NY, United States

**Keywords:** NLRP3 inflammasome, COVID-19, SARS-CoV-2, IL-1β, cytokine release syndrome (CRS), cytokine storm, coronavirus, acute respiratory distress syndrome (ARDS)

## Abstract

Severe acute respiratory syndrome coronavirus 2 (SARS-CoV-2) is a member of the genus *Betacoronavirus* within the family *Coronaviridae*. It is an enveloped single-stranded positive-sense RNA virus. Since December of 2019, a global expansion of the infection has occurred with widespread dissemination of coronavirus disease 2019 (COVID-19). COVID-19 often manifests as only mild cold-like symptomatology, but severe disease with complications occurs in 15% of cases. Respiratory failure occurs in severe disease that can be accompanied by a systemic inflammatory reaction characterized by inflammatory cytokine release. In severe cases, fatality is caused by the rapid development of severe lung injury characteristic of acute respiratory distress syndrome (ARDS). Although ARDS is a complication of SARS-CoV-2 infection, it is not viral replication or infection that causes tissue injury; rather, it is the result of dysregulated hyperinflammation in response to viral infection. This pathology is characterized by intense, rapid stimulation of the innate immune response that triggers activation of the Nod-like receptor family, pyrin domain-containing 3 (NLRP3) inflammasome pathway and release of its products including the proinflammatory cytokines IL-6 and IL-1β. Here we review the literature that describes the pathogenesis of severe COVID-19 and NLRP3 activation and describe an important role in targeting this pathway for the treatment of severe COVID-19.

## Introduction

Severe acute respiratory syndrome coronavirus 2 (SARS-CoV-2) is a member of the genus *Betacoronavirus* within the family *Coronaviridae*. It is an enveloped single-stranded positive-sense RNA virus (1). In December of 2019, the first cases of an atypical viral pneumonia were reported in Wuhan, China. Since that time, a global expansion of the infection has occurred with widespread dissemination of coronavirus disease 2019 (COVID-19) (2, 3). For most, the infection is mild with low-grade fever and cough, but 15% are associated with respiratory compromise. Severe cases result in acute respiratory distress syndrome (ARDS) with systemic inflammation in which lung injury is associated with release of inflammatory cytokines IL-6 and IL-1β (2, 4). The systemic inflammatory syndrome is characterized by dysregulated proinflammatory cytokine cascades triggered by an intense, rapid activation of the innate immune response. COVID-19 severity is associated with increased proinflammatory cytokines and chemokines and IL-6, specifically, is predictive of COVID-19 fatality (5). High levels of interleukin IL-1β and IL-6 were detected in autopsy tissues from SARS-CoV patients (6) and single cell RNA-seq analysis of peripheral blood in COVID-19 patients show increased subsets of CD14^+^ IL-1β-producing monocytes (7). A clear mechanism is not yet understood. The inflammatory basis underlying COVID-19 fatality renders development of immunoregulatory agents of paramount importance (8). There is significant literature implicating the Nod-like receptor family, pyrin domain-containing 3 (NLRP3) inflammasome, and cytokine release syndrome or cytokine storm in this pathogenesis (9–12). The NLRP3 inflammasome is an important cause of activation of the innate immune system to recognize pathogens, including viral infections (13, 14). SARS-CoV 3a protein activates the NLRP3 inflammasome in lipopolysaccharide-primed macrophages with 3a-mediated IL-1β secretion associated with K^+^ efflux and mitochondrial reactive oxygen species (15).

Individuals at risk for this inflammatory syndrome include those with hypertension, diabetes, cardiovascular disease, respiratory disease, and cancer (16, 17). It is not clear why individuals at risk include those with cardiovascular risk factors but may relate to the virology of SARS-CoV-2 infection. SARS-CoV uses the spike glycoprotein (S protein) on the surface of the virion to mediate viral membrane fusion (18). The S protein is a trimer that is cleaved into S1 and S2 subunits; S1 binds directly to the peptidase domain of angiotensin-converting enzyme 2 (ACE2) (19) to expose S2 to cleavage that enables fusion and entry (20). The physiological function of ACE2 in the cell is the maturation of angiotensin (Ang) which regulates blood pressure through vasoconstriction. Clinical literature based on the 2003 SARS-CoV epidemic suggested that the virus caused ACE2 downregulation and that lung injury may be improved by Angiotensive II Receptor Blocker (ARB) treatment (21, 22). Further literature implicates ACE2 signaling in NLRP3 activation in multiple settings. AngII can induce NLRP3 inflammasome activation in renal tubular epithelial cells (23), AngII induces pulmonary fibrosis which is attenuated by ACE2 (24), and NLRP3 inflammasome activation drives Ang II-induced vascular smooth muscle cell (VSMC) proliferation and vascular remodeling and hypertension (25, 26).

## COVID-19 Infection Clinical Syndrome

Individuals infected with SARS-CoV-2 can present with an array of clinical severity from asymptomatic through severe disease characterized by pneumonia requiring supplemental oxygen, and progression to acute respiratory distress syndrome (ARDS) with systemic inflammatory response syndrome (SIRS), shock and multiorgan dysfunction, coagulopathy, and death (27). Early symptoms can include shortness of breath, fever, and cough with increasing reports of loss of taste and smell (4, 17, 28–30). Individuals demonstrated to be at high risk of severe outcomes include those with advanced age, hypertension, cardiovascular disease, and diabetes mellitus (4, 29, 31, 32). Severe COVID-19 is associated with increased serum inflammatory cytokine levels including IL-1, IL-6, granulocyte-colony stimulating factor (G-CSF), interferon-γ inducible protein 10 (IP-10), and tumor necrosis factor-α (TNF-α) (5, 17, 33–36).

Overwhelming inflammatory cytokine secretion can result in ARDS through massive recruitment of immune cells leading to vascular leakage, fluid accumulation causing pulmonary edema, and resulting hypoxemia (37–39). Reports of patients with severe COVID-19 indicate that elevated levels of IL-1β and IL-6 are associated with elevated immune exhaustion and reduced T cell functional diversity (40). By contrast, individuals with COVID-19 who experience more mild disease have lower levels of IL-6, together with activated T lymphocytes and IgM SARS-CoV-2-binding antibodies (41). These observations indicate that a robust inflammatory cytokine response mediates severe disease while low inflammatory cytokine responses may be associated with an adaptive response that favors disease resolution. IL-1β is a key regulator of many chronic inflammatory diseases (42–49). Therefore, probing the role of IL-1β and its inhibition might lead to reduced inflammatory signaling, thus reducing lung injury in ARDS associated with severe COVID-19.

## NLRP3 Inflammasome Biology

The NLRP3 (NOD-, LRR-, and pyrin domain-containing protein 3) inflammasome consists of a sensor (NLRP3), an adaptor (ASC; also known as PYCARD), and an effector (caspase 1) (50). NLRP3 contains an amino-terminal pyrin domain (PYD), a central NACHT domain (domain present in NAIP, CIITA, HET-E, and TP1) and a carboxy-terminal leucine-rich repeat domain (LRR domain). The NACHT domain mediates ATPase function that is vital for NLRP3 self-association and function (51) and the LRR domains autoregulate through folding back onto the NACHT domain. ASC has two protein binding domains, an amino-terminal PYD and a carboxy-terminal caspase recruitment domain (CARD). NLRP3 can oligomerize between NACHT domains upon stimulation which leads to ASC recruitment through PYD–PYD interactions. The formation of multiple ASC filaments is referred to as an ASC speck (52–54). The assembled ASC complex can recruit caspase 1 to facilitate cleavage and activation.

Activation of the inflammasome is highly regulated and mediated by a two-step process in which first priming occurs and then activation occurs. Priming allows for transcription upregulation of the NLRP3 genes in response to recognition of pathogen-associated molecular patterns (PAMPs), such as lipopolysaccharides and viral RNA, or damage-associated molecular patterns (DAMPs), such as ATP and reactive oxygen species, through purine sensing receptors including P2RX7 (13, 14, 54–56). Engagement of PAMPS and/or DAMPS can activate pattern recognition receptors (PRRs) such as Toll-like receptors (TLRs) or nucleotide-binding oligomerization domain-containing protein 2 (NOD2). This leads to activation of nuclear factor-κB (NF-κB) activation and gene transcription (57). Priming also shifts oxidative phosphorylation to glycolysis in macrophages, resulting in stabilization of hypoxia-inducible factor 1α (HIF1α) and increase in *IL1B* gene transcription (58). Priming additionally induces post-translational modifications of the NLRP3 inflammasome which include ubiquitylation, phosphorylation, and sumoylation that stabilize the NLRP3 inflammasome in an auto-suppressed inactive, signal-competent, state (59).

After priming, NLRP3 inflammasome activation can occur in response to an array of pathogens or endogenous DAMPs. Multiple cellular signaling events can result in NLRP3 activation at the membrane, including efflux of potassium (K^+^) or chloride ions (Cl^−^), and flux of calcium ions (Ca^2+^) (60–70) as well as other cellular functions including lysosomal disruption, mitochondrial dysfunction, metabolic changes, and *trans*-Golgi disassembly (50).

NLRP3 activation can lead to pyroptosis, an inflammatory programmed cell death pathway that takes place in T lymphocytes (71). This inflammatory cell death is activated through gasdermin D (GSDMD) cleavage by caspase 1, 4, 5, and/or 11 and results in a series of cellular events including swelling of the cytoplasm, plasma membrane rupture, and consolidation of the nucleus with release of cytoplasmic contents into the extracellular space (72, 73). GSDMD contains an amino-terminal cell death domain (GSDMD^Nterm^) which is exposed through caspase cleavage to bind phosphatidylinositol phosphates and phosphatidylserine in the cell membrane, inserting into the plasma membrane and forming a pore that kills the cell from within (74, 75). Additionally, GSDMD can mediate IL-1β and IL-18 secretion (76, 77) and this occurs both through pathways dependent and independent of NLRP3 signaling.

Cell death is an important cause of pathogenesis in viral infections. HIV-1 infection is associated with programmed cell death through pyroptosis in bystander cells (78–82) and represents an important mechanism of NLRP3 inflammasome-mediated immune cell depletion. Programmed cell death through multiple mechanisms has been reported in coronavirus infections as an important mechanism of viral pathogenesis (83–88).

## The NLRP3 Inflammasome in Coronavirus Pathogenesis

There are numerous studies that implicate the NLRP3 inflammasome and IL-1β in mediating inflammation during lung injury and ARDS (39, 89, 90). Bronchoalveolar fluid and plasma in patients with ARDS have elevated IL-1β levels compared to healthy controls (91–94) and is associated with worse clinical outcomes. In other coronavirus infections including MERS-CoV and SARS-CoV, patients with ARDS had high levels of IL-1β, IL-6, and IL-8 (6, 95–97). In other respiratory viral infections such as influenza, high levels of IL-1β have been detected in bronchoalveolar fluid and plasma from patients with lung injury (91–94, 98–101). Furthermore, animal studies in which mice deficient in components of the inflammasome have reduced lung injury and enhanced survival with influenza infection (45, 102). In pharmacologic studies in which IL-1β or IL-1R was antagonized, influenza associated lung injury was reduced (103, 104). Taken together, IL-1β appears to play a key role in acute lung injury with respiratory viral infections and pharmacologic targeting of this pathway represents an important area of intervention.

Injury of type II alveolar epithelial cells expressing ACE2 leads to NLRP3 inflammasome activation (14, 15, 105). The acute immune response to SARS-CoV-2 infection is largely driven by inflammatory alveolar and monocyte-derived macrophages that are activated by PAMPs and DAMPs released by infected, apoptotic pneumocytes (11, 106–108). TNF-α and IL-1β secreted by alveolar macrophages initiate the acute proinflammatory cascade immediately following infection. The secretion of these cytokines induces cell death and damage, PAMP/DAMP production, immune cell recruitment, and widespread NLRP3 activation, establishing a proinflammatory positive feedback cascade (11, 106, 108–110). More recently, Blanco-Melo et al. demonstrated that SARS-CoV-2 infection of primary human bronchial epithelial cells resulted in expression of multiple cytokines and chemokines including TNF-α, IL-6, and IL-1β (111).

This localized inflammatory cell death extends to the vasculature, inducing the leakage, edema, and pneumonia characteristic of COVID-19 (11, 108, 109). It is important to note that the onset of this pathological immune response is characterized not by systemic inflammation, but by a hyperinflammatory microenvironment localized to the site of tissue injury. As the inflammatory cascade progresses, IL-1β, and TNF-α induce the secretion of additional NLRP3 cytokines such as IL-6 which can subsequently be observed in the peripheral blood due to the loss of vascular integrity (11, 107–110, 112, 113). The kinetics of the inflammatory response are essential to effective clinical practice—circulating biomarkers such as IL-6 may prove useful to predicting outcomes and informing immunomodulatory treatment decisions (31, 33, 114–116).

The rapid decline of COVID-19 patients coincides with an abrupt shift from the NLRP3 cytokine storm to a compensatory immunosuppressive state (5, 107). This repair and recovery-oriented phase is characterized by production of IL-10, polarization of macrophages to the anti-inflammatory M2 state, suppression of NLRP3, and recruitment of fibroblasts and platelets. The accumulation of fibroblasts and M2 macrophages in the lung initiates the deposition of collagen and construction of the extracellular matrices that characterize ARDS fibrosis (11, 108, 117). M2 macrophages and other markers of this pro-fibrotic, anti-inflammatory environment have been detected in the bronchioalveolar fluid of severe COVID-19 patients (117, 118).

Unique to SARS-CoV and SARS-CoV-2 is the downmodulation of the ACE2 receptor. SARS-CoV entry has been reported to be dependent on TNF-α converting enzyme and coupled to the release of TNF-α from the cell membrane (110). TNF-α, specifically, has been shown to act as an alternative toll-like receptor (TLR) agonist that may increase the sensitivity and longevity of NLRP3 activation (113, 119). Downregulation of ACE2 is associated with both SARS-CoV and SARS-CoV-2 disease severity (21, 120, 121); this contrasts with a minimally symptomatic coronavirus strain, HCoV-NL63, that utilizes but does not cleave or downmodulate the ACE2 receptor (122). The overproduction of TNF-α in COVID-19 may preferentially activate the NLRP3 inflammasome relative to other immunological pathways. These observations warrant further investigation into the mechanisms by and extent to which TNF-α acts as a significant modulator of severe COVID-19.

The SARS-CoV genome encodes 3 ion channel proteins: E, open reading frame 3a (ORF3a), and ORF8a in which E and ORF3a are required for both replication and virulence (87, 109, 123–126). In addition to the canonical NLRP3 activation pathway by PAMPs and DAMPs, the E, 3a, and 8b proteins of SARS-CoV function as NLRP3 agonists (84, 107, 109, 123, 127); many of these sequences are conserved in SARS-CoV-2 and likely play a role in inflammatory pathogenesis (107, 128). The SARS-CoV E, 3a, and 8b proteins are all reported to induce NLRP3 activation and IL-1β release in LPS-primed macrophage models (15, 127). A wide variety of mechanisms have been proposed for this NLRP3 agonism including E-, 3a-, and 8b-induced viroporin activity, interferon antagonism, membrane-bound organelle stress, reactive oxygen species production, and direct binding to and regulation of inflammasome components such as caspase 1, NLRP3, and NF-κB (15, 86, 107, 109, 112, 123, 127). There are multiple pathways by which SARS-CoV triggers NLRP3 activation which have yet to be characterized and are likely influenced by cell type and the extracellular microenvironment (15, 84, 86, 88, 107).

Notably, the NLRP3-implicated ORFs 3a and 8 are the primary sites driving genetic diversification of SARS-CoV-2. ORF3a, specifically, is the only gene undergoing diversifying mutations that are predicted to exhibit altered phenotypes (84, 113, 127, 129). Ongoing mutations in ORF8 are particularly concerning, as a 29-nt deletion of the SARS-CoV genome is suspected to have increased the pathogenicity of the virus during the SARS-CoV epidemic by antagonizing interferon, increasing viral titers, and agonizing NLRP3 (127, 130). The uniquely low homology between SARS-CoV-2 and SARS-CoV ORFs 3a and 8 may play a role in the differences in virulence and pathogenesis between these two related viral infections (107, 131). Defining the inflammatory activities of these two proteins is therefore critical to predictive monitoring and modeling of novel SARS-CoV-2 strain emergence.

Genetic variations in host inflammasome pathways may also influence disease outcome. Mutations in the LRR domain of bat NLRP3 mediate an overall dampened NLRP3 response to agonists (85). In the context of coronavirus infections, MERS-CoV does not induce clinical disease in bats despite high viral titers; this appears to be mediated by NLRP3 (85). Interestingly, SARS-CoV ORF8b is reported to activate NLRP3 via direct binding to the LRR domain, suggesting a mechanism of coronavirus-induced NLRP3 activation and further indicating therapeutic potential for NLRP3 immunomodulatory agents (127). Defining these mechanisms should be a focus of SARS-CoV-2 research so as to identify targeted therapeutics such as those summarized in Table 1.

**Table 1:** NLRP3 inflammasome-targeted therapeutics in development.

**Development stage**	**Drug name**	**Company**	**Mechanism of action**	**Reference(s)**
Preclinical	N/A	Ardan ImmunoPharma	Small-molecule activators and inhibitors of the TMEM176B ion channel, which is an inhibitor of the inflammasome	(132, 133)
	N/A	Genentech	NLRP3 inhibitors acquired from Jecure Therapeutics	(134)
	N/A	IFM Therapeutics	Small-molecule inhibitors of the NLRP1, NLRP6, NLRP10, and NLRC4 inflammasomes	(134)
	N/A	NodThera	Small-molecule NLRP3 inhibitors expected to begin clinical studies this year	(134)
	IC 100	ZyVersa Therapeutics	Antibody inhibitors of the inflammasome protein ASC	(135)
Phase I	N/A	Bristol-Myers Squibb	NLRP3 activators for cancer immunotherapy acquired from IFM Therapeutics	(134)
	CRID3 (CP-456, 773, MCC950)	Pfizer	Selective NLRP3 inhibitor	(134, 136–138)
	Inzomelid (also Somalix)	Inflazome	Small-molecule NLRP3 inhibitors	(134, 139)
	IFM-2427	Novartis	Small-molecule NLRP3 inhibitors acquired from IFM Therapeutics and developed in-house	(135)
Phase II	Dapansutrile (OLT1177)	Olatec Therapeutics	Small-molecule NLRP3 inhibitors	(140–143)
	Canakinumab	Novartis	IL-1β-neutralizing antibody	(144, 145)
	Anakinra	Sobi	Recombinant IL-1 receptor antagonist	(146, 147)
	Rilonacept	Regeneron	Decoy receptor that binds IL-1β and IL-1α	(147–149)
	Gevokizumab	XOMA	Decreases the binding affinity of IL-1β for the IL-1 receptor	(150–152)

## The NLRP3 Inflammasome in Cytokine Release Syndromes

Cytokine release syndrome (CRS) is a systemic inflammatory response that can be triggered by a number of stimuli including drugs and infections (153, 154). The term was originally coined in response to administration of anti-T-cell antibody muromonab-CD3 (OKT3) to solid organ transplant patients who experienced an idiosyncratic cytokine storm following treatment (155, 156). A number of other drugs have stimulated similar infusion reactions including antibody-based therapies (157–164) and cancer therapeutics (165, 166). Other reported stimuli for the development of CRS include haploidentical donor stem cell transplantation, graft-vs.-host disease (167, 168), and respiratory viral infections including influenza (11, 169). Most recently, new classes of immunotherapeutic agents are used in a variety of hematologic malignancies including bispecific antibody constructs and chimeric antigen receptor (CAR) T cell therapies.

In response to these stimuli, patients experience robust cytokine-mediated response that is associated with fever, hypotension and hypoxemia. The syndrome can be mild and resolve spontaneously or can progress to persistent high-grade fevers, vasodilatory shock with hemodynamic instability, severe hypoxemia requiring mechanical ventilation. This can be associated with end-organ damage including liver injury, cardiac ischemia, clotting dysfunction, kidney dysfunction, and hemophagocytic lymphohistiocytosis/macrophage activation syndrome (HLH/MAS) (154). The timing of onset is unpredictable, between 1 day to 2 months after exposure (170).

**Figure 1:**
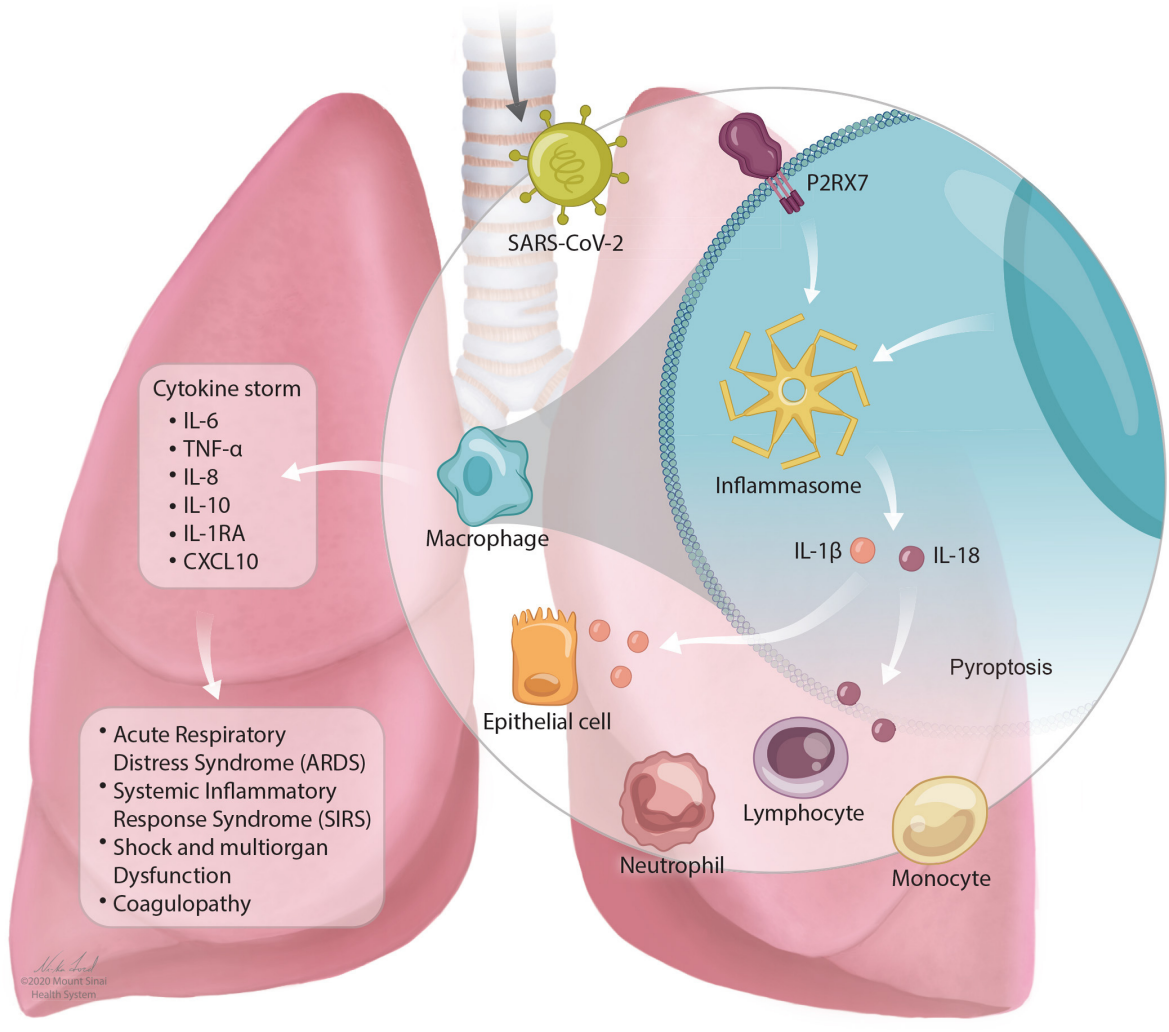
The NLRP3 inflammasome mediates lung inflammation in SARS-CoV-2 infection. SARS-CoV-2 is inhaled into the airway and mediates activation of the P2RX7 receptor by release of extracellular ATP. P2RX7 signaling can lead to NLRP3 activation through direct or indirect activation in activated macrophages. Activation of the NLRP3 inflammasome drives the secretion of IL-1β and IL-18 which can result in pyroptosis (programmed cell death). Activation of immune cell subsets, largely through activated macrophages, results in a cascade of massive inflammatory cytokine activation including IL-6, TNF-α, IL-8, IL-10, IL-1RA, and CXCL10 that lead to acute lung injury with acute respiratory distress syndrome, systemic inflammatory response syndrome (SIRS), shock and multiorgan dysfunction, and coagulopathy.

In SARS-CoV-2 infection, a cytokine storm occurs that has similar features to CRS as described above. Individuals with severe COVID-19 with cytokine storm have elevated systemic inflammatory biomarkers including C-reactive protein, D-dimer, ferritin (3, 115, 171–173). Patients experience a dysfunctional immune response characterized by high levels of plasma cytokines including IL-6, TNF-α, IL-8, IL-10, IL-1RA, and CXCL10 (4, 117). IL-6 levels increase over time higher in those who die of the infection compared to those who survive (27). The stimulation of inflammatory cytokines, largely through activated macrophages, leads to acute lung injury, acute respiratory distress syndrome, systemic inflammatory response syndrome (SIRS), shock and multiorgan dysfunction, and coagulopathy (117). This is described in Figure 1.

Individuals with severe COVID-19 have developed a coagulopathy which is associated with reduced platelet count, increased levels of fibrin degradation productions (D-dimer), and increased microthrombi in lungs, brain, kidney, and extremities (174–176). The NLRP3 inflammasome may play a key role in mediating this coagulopathy. Activated macrophages undergoing NLRP3 inflammasome activation release tissue factor which initiates coagulation (177, 178), regulation of platelet integrins (179, 180), and through hypoxia-inducible factor 1-alpha (HIF-1α) (181). Whether NLRP3 inflammasome activation as a mediator of coagulopathy is an area of great interest for future investigation.

## NLRP3-Targeted Therapeutics

Experimental therapeutics assessed *in vitro* and *in vivo* have provided further insight into the role of NLRP3 in mediating SARS-CoV pathogenicity. In bone marrow-derived macrophages, a mitochondrial antioxidant reduced IL-1β secretion induced by SARS-CoV 3a and E proteins (15). In SARS-CoV-infected mice, the NF-κB antagonists CAPE, resveratrol, Bay11-7082, and parthenolide improved survival and reduced proinflammatory cytokine levels in the lungs (182). Depletion of inflammatory macrophages also mitigated SARS-CoV-associated inflammatory lung pathology in mice without impacting viral load (108). These reports elucidate molecular and clinical inflammatory phenotypes that appear to parallel those seen in COVID-19 and should be used to inform novel therapeutic development and pathogenesis studies.

Cross-regulation between type I interferon (IFN-I) and the NLRP3 inflammasome is implicated in the abrupt proinflammatory response to immunosuppressive switch characteristic of SARS and COVID-19 ARDS through an undefined mechanism (5, 107). Early IFN-I administration may therapeutically regulate NLRP3 and has been shown to abrogate clinical symptomatology in SARS-CoV-infected macaques (112) and mice (108). Dual corticosteroid-IFN-I treatment appeared to improve outcomes in a small-cohort SARS-CoV trial (183, 184). The therapeutic impact observed in mice, macaques, and humans in each setting occurred despite unchanged viral loads (108, 112, 183, 184).

Both IL-6R and IL-1 receptor blocking agents have been used for the treatment of CRS (185, 186). Tocilizumab, an IL-6R blocking antibody has been used to treat severe CRS (187, 188) in the setting of CAR-T cell therapy and in the setting of SARS-CoV-2 infection (5). Similarly, the IL-1 receptor antagonist anakinra improves CAR-T cell therapy CRS outcomes and also significantly increases survival of SARS-CoV-infected mice with hyperactive NLRP3 inflammasomes (186, 189, 190). In a retrospective cohort analysis, intravenous administration of high-dose anakinra increased survival and clinical improvement in COVID-19 patients with ARDS (191). Evidence from CAR-T-induced CRS suggests parallels to the COVID-19 inflammatory response that would suggest that targeting IL-1β would reduce the inflammatory signaling that mediates lung injury, ARDS, and mortality. Table 1 shows a list of agents in various stages of development that target the NLRP3 inflammasome.

Therapeutics targeting IL-1β and the NLRP3 inflammasome pathway have similarly been employed and efficacious in the context of cardiovascular disease. The NLRP3 inhibitors arglabin and MCC950 reduced IL-1β plasma levels and decreased atherosclerotic lesion size (48, 192). IL-1β neutralizing antibodies and anakinra showed reduced cardiac hypertrophy and myocardial dysfunction post-MI (193–195). The CANTOS trial randomized patients with past MI and elevated hsCRP to receive canakinumab, a monoclonal antibody targeting IL-1β and found a 15% reduction in major CV events (144).

## Conclusions

In sum, COVID-19 causes an array of disease manifestations, the most severe of which is mediated by a massive inflammatory response that appears to occur through stimulation of the NLRP3 inflammasome. Direct data linking the NLRP3 inflammasome and SARS-CoV-2 infection are limited given the recent onset of this new pathogen and its global impact. The pathogenesis of this infection and cytokine storm, mirrors many of those features observed in cardiovascular disease, HIV-1 pathogenesis, and SARS-CoV. For this reason, it is of value to contextualize what is already known about the NLRP3 as a mediator of inflammatory signaling to inform future studies of pathogenesis and therapeutic development given the urgent need for drug discovery.

Significant evidence supports the role of IL-1β and NLRP3-dependent inflammasome activation in the pathogenesis of acute lung injury. An abundance of literature supports targeting this pathway in the development of therapeutic strategies. In consideration of direct acting anti-viral agents, viral load appears non- or minimally consequential in determining SARS-CoV and SARS-CoV-2 disease outcomes. When tested in the context of SARS-CoV infection, treatments targeting NLRP3 pathway components including NF-κB, inflammatory macrophages, and IFN-I all demonstrated significant efficacy despite unchanged viral titers their respective human, murine, macaque, and/or *in vitro* models (5, 35, 106, 107, 196). In COVID-19 clinical trials, hydroxychloroquine demonstrated antiviral activity (197, 198), yet without demonstrated clinical benefit (199–201). The known role of NLRP3 in hyperinflammatory ARDS and CRS, documented NLRP3 involvement in MERS-CoV and SARS-CoV severity, and apparent efficacy of anti-NLRP3 therapeutics in SARS-CoV and SARS-CoV-2 clinical trials and animal models strongly indicate that NLRP3 is a central mediator of severe COVID-19. The potential central role of NLRP3 in severe COVID-19 necessitates investigation into the therapeutic targeting of the NLRP3 inflammasome.

Timing of therapy is critical as once individuals develop ARDS, the chances of improved outcomes with therapy are severely reduced. Targeted therapy for individuals with moderate disease before the development of respiratory failure will be critical. There is an urgent need to develop therapeutics that improve patient outcomes in severe COVID-19. Therefore, targeting this pathway through existing available therapeutic options would represent an important and viable approach to reducing SARS-CoV-2-induced inflammatory cytokine signaling and immediately improve patient outcomes.

## Author Contributions

All authors listed have made a substantial, direct and intellectual contribution to the work, and approved it for publication.

## Conflict of Interest

The authors declare that the research was conducted in the absence of any commercial or financial relationships that could be construed as a potential conflict of interest.

## References

[1] Coronaviridae Study Group of the International Committee on Taxonomy of Viruses. The species severe acute respiratory syndrome-related coronavirus: classifying 2019-nCoV and naming it SARS-CoV-2. Nat Microbiol. (2020) 5:536–44. 10.1038/s41564-020-0695-z 32123347 PMC7095448

[2] Zhu N Zhang D Wang W Li X Yang B Song J. A novel coronavirus from patients with pneumonia in China 2019. N Engl J Med. (2020) 382:727–33. 10.1056/NEJMoa2001017 31978945 PMC7092803

[3] Zhou P Yang XL Wang XG Hu B Zhang L Zhang W. A pneumonia outbreak associated with a new coronavirus of probable bat origin. Nature. (2020) 579:270–3. 10.1038/s41586-020-2012-7 32015507 PMC7095418

[4] Huang C Wang Y Li X Ren L Zhao J Hu Y. Clinical features of patients infected with 2019 novel coronavirus in Wuhan, China. Lancet. (2020) 395:497–506. 10.1016/S0140-6736(20)30183-5 31986264 PMC7159299

[5] Mehta P McAuley DF Brown M Sanchez E Tattersall RS Manson JJ. COVID-19: consider cytokine storm syndromes and immunosuppression. Lancet. (2020) 395:1033–1034. 10.1016/S0140-6736(20)30628-0 32192578 PMC7270045

[6] He L Ding Y Zhang Q Che X He Y Shen H. Expression of elevated levels of pro-inflammatory cytokines in SARS-CoV-infected ACE2+ cells in SARS patients: relation to the acute lung injury and pathogenesis of SARS. J Pathol. (2006) 210:288–97. 10.1002/path.2067 17031779 PMC7167655

[7] Wen W Su W Tang H Le W Zhang X Zheng Y. Immune cell profiling of COVID-19 patients in the recovery stage by single-cell sequencing. Cell Discov. (2020) 6:31. 10.1038/s41421-020-0168-9 32377375 PMC7197635

[8] Vabret N Samstein R Fernandez N Merad M Project SIR The Sinai Immunology Review Project, Trainees & Faculty. Advancing scientific knowledge in times of pandemics. Nat Rev Immunol. (2020) 20:338. 10.1038/s41577-020-0319-0 32327718 PMC7187143

[9] Lin L Xu L Lv W Han L Xiang Y Fu L. An NLRP3 inflammasome-triggered cytokine storm contributes to Streptococcal toxic shock-like syndrome (STSLS). PLoS Pathog. (2019) 15:e1007795. 10.1371/journal.ppat.1007795 31170267 PMC6553798

[10] Sendler M van den Brandt C Glaubitz J Wilden A Golchert J Weiss FU. NLRP3 inflammasome regulates development of systemic inflammatory response and compensatory anti-inflammatory response syndromes in mice with acute pancreatitis. Gastroenterology. (2020) 158:253–69.e14. 10.1053/j.gastro.2019.09.040 31593700

[11] Tisoncik JR Korth MJ Simmons CP Farrar J Martin TR Katze MG. Into the eye of the cytokine storm. Microbiol Mol Biol Rev. (2012) 76:16–32. 10.1128/MMBR.05015-11 22390970 PMC3294426

[12] Chousterman BG Swirski FK Weber GF. Cytokine storm and sepsis disease pathogenesis. Semin Immunopathol. (2017) 39:517–28. 10.1007/s00281-017-0639-8 28555385

[13] Bauernfeind F Ablasser A Bartok E Kim S Schmid-Burgk J Cavlar T. Inflammasomes: current understanding and open questions. Cell Mol Life Sci. (2011) 68:765–83. 10.1007/s00018-010-0567-4 21072676 PMC11114650

[14] Zhao C Zhao W. NLRP3 inflammasome-a key player in antiviral responses. Front Immunol. (2020) 11:211. 10.3389/fimmu.2020.00211 32133002 PMC7040071

[15] Chen IY Moriyama M Chang MF Ichinohe T. Severe acute respiratory syndrome coronavirus viroporin 3a activates the NLRP3 inflammasome. Front Microbiol. (2019) 10:50. 10.3389/fmicb.2019.00050 30761102 PMC6361828

[16] Li JY You Z Wang Q Zhou ZJ Qiu Y Luo R. The epidemic of 2019-novel-coronavirus (2019-nCoV) pneumonia and insights for emerging infectious diseases in the future. Microbes Infect. (2020) 22:80–5. 10.1016/j.micinf.2020.02.002 32087334 PMC7079563

[17] Wang D Hu B Hu C Zhu F Liu X Zhang J. Clinical characteristics of 138 hospitalized patients with 2019 novel coronavirus-infected pneumonia in Wuhan, China. JAMA. (2020) 323:1061–9. 10.1001/jama.2020.1585 32031570 PMC7042881

[18] Simmons G Reeves JD Rennekamp AJ Amberg SM Piefer AJ Bates P. Characterization of severe acute respiratory syndrome-associated coronavirus (SARS-CoV) spike glycoprotein-mediated viral entry. Proc Natl Acad Sci USA. (2004) 101:4240–5. 10.1073/pnas.0306446101 15010527 PMC384725

[19] Li F Li W Farzan M Harrison SC. Structure of SARS coronavirus spike receptor-binding domain complexed with receptor. Science. (2005) 309:1864–8. 10.1126/science.1116480 16166518

[20] Millet JK Whittaker GR. Host cell proteases: critical determinants of coronavirus tropism and pathogenesis. Virus Res. (2015) 202:120–34. 10.1016/j.virusres.2014.11.021 25445340 PMC4465284

[21] Kuba K Imai Y Rao S Gao H Guo F Guan B. A crucial role of angiotensin converting enzyme 2 (ACE2) in SARS coronavirus-induced lung injury. Nat Med. (2005) 11:875–9. 10.1038/nm1267 16007097 PMC7095783

[22] Patel AB Verma A COVID-19 and angiotensin-converting enzyme inhibitors and angiotensin receptor blockers: what is the evidence? JAMA. (2020) 323:1769–70. 10.1001/jama.2020.4812 32208485

[23] Wen Y Liu Y Tang T Lv L Liu H Ma K. NLRP3 inflammasome activation is involved in Ang II-induced kidney damage via mitochondrial dysfunction. Oncotarget. (2016) 7:54290–302. 10.18632/oncotarget.11091 27509058 PMC5342342

[24] Sun NN Yu CH Pan MX Zhang Y Zheng BJ Yang QJ. Mir-21 mediates the inhibitory effect of Ang (1-7) on AngII-induced NLRP3 inflammasome activation by targeting Spry1 in lung fibroblasts. Sci Rep. (2017) 7:14369. 10.1038/s41598-017-13305-3 29084974 PMC5662719

[25] Ren XS Tong Y Ling L Chen D Sun HJ Zhou H. NLRP3 gene deletion attenuates angiotensin ii-induced phenotypic transformation of vascular smooth muscle cells and vascular remodeling. Cell Physiol Biochem. (2017) 44:2269–80. 10.1159/000486061 29262411

[26] Sun HJ Ren XS Xiong XQ Chen YZ Zhao MX Wang JJ. NLRP3 inflammasome activation contributes to VSMC phenotypic transformation and proliferation in hypertension. Cell Death Dis. (2017) 8:e3074. 10.1038/cddis.2017.470 28981106 PMC5680591

[27] Zhou F Yu T Du R Fan G Liu Y Liu Z. Clinical course and risk factors for mortality of adult inpatients with COVID-19 in Wuhan, China: a retrospective cohort study. Lancet. (2020) 395:1054–62. 10.1016/S0140-6736(20)30566-3 32171076 PMC7270627

[28] Team CC-R. Characteristics of health care personnel with COVID-19 - United States, February 12-April 9, 2020. MMWR Morb Mortal Wkly Rep. (2020) 69:477–81. 10.15585/mmwr.mm6915e6 32298247 PMC7755055

[29] Guan WJ Ni ZY Hu Y Liang WH Ou CQ He JX. Clinical characteristics of coronavirus disease 2019 in China. N Engl J Med. (2020) 382:1708–20. 10.1056/NEJMoa2002032 32109013 PMC7092819

[30] Zhao D Yao F Wang L Zheng L Gao Y Ye J. A comparative study on the clinical features of COVID-19 pneumonia to other pneumonias. Clin Infect Dis. (2020) ciaa247. 10.1093/cid/ciaa247 32161968 PMC7108162

[31] Zhu Z Cai T Fan L Lou K Hua X Huang Z. Clinical value of immune-inflammatory parameters to assess the severity of coronavirus disease 2019. Int J Infect Dis. (2020)95:332–9. 10.1016/j.ijid.2020.04.041 32334118 PMC7195003

[32] Chen T Wu D Chen H Yan W Yang D Chen G. Clinical characteristics of 113 deceased patients with coronavirus disease 2019: retrospective study. BMJ. (2020) 368:m1091. 10.1136/bmj.m1091 32217556 PMC7190011

[33] Conti P Ronconi G Caraffa A Gallenga CE Ross R Frydas I. Induction of pro-inflammatory cytokines (IL-1 and IL-6) and lung inflammation by Coronavirus-19 (COVI-19 or SARS-CoV-2): anti-inflammatory strategies. J Biol Regul Homeost Agents. (2020) 34:1. 10.23812/CONTI-E 32171193

[34] Giamarellos-Bourboulis EJ Netea MG Rovina N Akinosoglou K Antoniadou A Antonakos N. Complex immune dysregulation in COVID-19 patients with severe respiratory failure. Cell Host Microbe. (2020) 27:992–1000.e3. 10.1016/j.chom.2020.04.009 32320677 PMC7172841

[35] Zhang C Wu Z Li JW Zhao H Wang GQ. The cytokine release syndrome (CRS) of severe COVID-19 and Interleukin-6 receptor (IL-6R) antagonist Tocilizumab may be the key to reduce the mortality. Int J Antimicrob Agents. (2020) 55:105954. 10.1016/j.ijantimicag.2020.105954 32234467 PMC7118634

[36] Amlani A Barber C Fifi-Mah A Monzon J. Successful treatment of cytokine release syndrome with IL-6 blockade in a patient transitioning from immune-checkpoint to MEK/BRAF inhibition: a case report and review of literature. Oncologist. (2020) 25:1–4. 10.1634/theoncologist.2020-0194 32337758 PMC7356700

[37] Leff JA Baer JW Bodman ME Kirkman JM Shanley PF Patton LM. Interleukin-1-induced lung neutrophil accumulation and oxygen metabolite-mediated lung leak in rats. Am J Physiol. (1994) 266:L2–8. 10.1152/ajplung.1994.266.1.L2 8304466

[38] Olman MA White KE Ware LB Simmons WL Benveniste EN Zhu S. Pulmonary edema fluid from patients with early lung injury stimulates fibroblast proliferation through IL-1 beta-induced IL-6 expression. J Immunol. (2004) 172:2668–77. 10.4049/jimmunol.172.4.2668 14764742

[39] Ganter MT Roux J Miyazawa B Howard M Frank JA Su G. Interleukin-1beta causes acute lung injury via alphavbeta5 and alphavbeta6 integrin-dependent mechanisms. Circ Res. (2008) 102:804–12. 10.1161/CIRCRESAHA.107.161067 18276918 PMC2739091

[40] Zheng HY Zhang M Yang CX Zhang N Wang XC Yang XP. Elevated exhaustion levels and reduced functional diversity of T cells in peripheral blood may predict severe progression in COVID-19 patients. Cell Mol Immunol. (2020) 17:541–3. 10.1038/s41423-020-0401-3 32203186 PMC7091621

[41] Thevarajan I Nguyen THO Koutsakos M Druce J Caly L van de Sandt CE. Breadth of concomitant immune responses prior to patient recovery: a case report of non-severe COVID-19. Nat Med. (2020) 26:453–5. 10.1038/s41591-020-0819-2 32284614 PMC7095036

[42] Toldo S Mezzaroma E Bressi E Marchetti C Carbone S Sonnino C. Interleukin-1β blockade improves left ventricular systolic/diastolic function and restores contractility reserve in severe ischemic cardiomyopathy in the mouse. J Cardiovasc Pharmacol. (2014) 64:1–6. 10.1097/FJC.0000000000000106 25006675

[43] Mantovani A Dinarello CA Molgora M Garlanda C. Interleukin-1 and related cytokines in the regulation of inflammation and immunity. Immunity. (2019) 50:778–95. 10.1016/j.immuni.2019.03.012 30995499 PMC7174020

[44] Zhao C Gu Y Zeng X Wang J. NLRP3 inflammasome regulates Th17 differentiation in rheumatoid arthritis. Clin Immunol. (2018) 197:154–60. 10.1016/j.clim.2018.09.007 30236770

[45] Zhang H Luo J Alcorn JF Chen K Fan S Pilewski J. AIM2 inflammasome is critical for influenza-induced lung injury and mortality. J Immunol. (2017) 198:4383–93. 10.4049/jimmunol.1600714 28424239 PMC5439025

[46] Yang CA Huang ST Chiang BL. Association of NLRP3 and CARD8 genetic polymorphisms with juvenile idiopathic arthritis in a Taiwanese population. Scand J Rheumatol. (2014) 43:146–52. 10.3109/03009742.2013.834962 24295199

[47] Walsh JG Reinke SN Mamik MK McKenzie BA Maingat F Branton WG. Rapid inflammasome activation in microglia contributes to brain disease in HIV/AIDS. Retrovirology. (2014) 11:35. 10.1186/1742-4690-11-35 24886384 PMC4038111

[48] van der Heijden T Kritikou E Venema W van Duijn J van Santbrink PJ Slutter B. NLRP3 inflammasome inhibition by MCC950 reduces atherosclerotic lesion development in apolipoprotein E-deficient mice-brief report. Arterioscler Thromb Vasc Biol. (2017) 37:1457–61. 10.1161/ATVBAHA.117.309575 28596375

[49] Tan HY Yong YK Shankar EM Paukovics G Ellegard R Larsson M. Aberrant inflammasome activation characterizes tuberculosis-associated immune reconstitution inflammatory syndrome. J Immunol. (2016) 196:4052–63. 10.4049/jimmunol.1502203 27076678

[50] Swanson KV Deng M Ting JP. The NLRP3 inflammasome: molecular activation and regulation to therapeutics. Nat Rev Immunol. (2019) 19:477–89. 10.1038/s41577-019-0165-0 31036962 PMC7807242

[51] Duncan JA Bergstralh DT Wang Y Willingham SB Ye Z Zimmermann AG. Cryopyrin/NALP3 binds ATP/dATP, is an ATPase, and requires ATP binding to mediate inflammatory signaling. Proc Natl Acad Sci USA. (2007) 104:8041–6. 10.1073/pnas.0611496104 17483456 PMC1876568

[52] Schmidt FI Lu A Chen JW Ruan J Tang C Wu H. A single domain antibody fragment that recognizes the adaptor ASC defines the role of ASC domains in inflammasome assembly. J Exp Med. (2016) 213:771–90. 10.1084/jem.20151790 27069117 PMC4854733

[53] Lu A Magupalli VG Ruan J Yin Q Atianand MK Vos MR. Unified polymerization mechanism for the assembly of ASC-dependent inflammasomes. Cell. (2014) 156:1193–206. 10.1016/j.cell.2014.02.008 24630722 PMC4000066

[54] Ruland J. Inflammasome: putting the pieces together. Cell. (2014) 156:1127–9. 10.1016/j.cell.2014.02.038 24630715

[55] Skeldon AM Faraj M Saleh M. Caspases and inflammasomes in metabolic inflammation. Immunol Cell Biol. (2014) 92:304–13. 10.1038/icb.2014.5 24518981

[56] Di Virgilio F Dal Ben D Sarti AC Giuliani AL Falzoni S. The P2X7 receptor in infection and inflammation. Immunity. (2017) 47:15–31. 10.1016/j.immuni.2017.06.020 28723547

[57] Bauernfeind FG Horvath G Stutz A Alnemri ES MacDonald K Speert D. Cutting edge: NF-kappaB activating pattern recognition and cytokine receptors license NLRP3 inflammasome activation by regulating NLRP3 expression. J Immunol. (2009) 183:787–91. 10.4049/jimmunol.0901363 19570822 PMC2824855

[58] Tannahill GM Curtis AM Adamik J Palsson-McDermott EM McGettrick AF Goel G. Succinate is an inflammatory signal that induces IL-1β through HIF-1α. Nature. (2013) 496:238–42. 10.1038/nature11986 23535595 PMC4031686

[59] Shim DW Lee KH. Posttranslational regulation of the NLR family pyrin domain-containing 3 inflammasome. Front Immunol. (2018) 9:1054. 10.3389/fimmu.2018.01054 29868015 PMC5968104

[60] Perregaux D Gabel CA. Interleukin-1 beta maturation and release in response to ATP and nigericin. Evidence that potassium depletion mediated by these agents is a necessary and common feature of their activity. J Biol Chem. (1994) 269:15195–203. 8195155

[61] Surprenant A Rassendren F Kawashima E North RA Buell G. The cytolytic P2Z receptor for extracellular ATP identified as a P2X receptor (P2X7). Science. (1996) 272:735–8. 10.1126/science.272.5262.735 8614837

[62] Samways DS Li Z Egan TM. Principles and properties of ion flow in P2X receptors. Front Cell Neurosci. (2014) 8:6. 10.3389/fncel.2014.00006 24550775 PMC3914235

[63] Di A Xiong S Ye Z Malireddi RKS Kometani S Zhong M. The TWIK2 potassium efflux channel in macrophages mediates NLRP3 inflammasome-induced inflammation. Immunity. (2018) 49:56–65.e4. 10.1016/j.immuni.2018.04.032 29958799 PMC6051907

[64] Triantafilou K Hughes TR Triantafilou M Morgan BP. The complement membrane attack complex triggers intracellular Ca^2+^ fluxes leading to NLRP3 inflammasome activation. J Cell Sci. (2013) 126:2903–13. 10.1242/jcs.124388 23613465

[65] Muñoz-Planillo R Kuffa P Martínez-Colón G Smith BL Rajendiran TM Núñez G. K^+^ efflux is the common trigger of NLRP3 inflammasome activation by bacterial toxins and particulate matter. Immunity. (2013) 38:1142–53. 10.1016/j.immuni.2013.05.016 23809161 PMC3730833

[66] Murakami T Ockinger J Yu J Byles V McColl A Hofer AM. Critical role for calcium mobilization in activation of the NLRP3 inflammasome. Proc Natl Acad Sci USA. (2012) 109:11282–7. 10.1073/pnas.1117765109 22733741 PMC3396518

[67] Lee GS Subramanian N Kim AI Aksentijevich I Goldbach-Mansky R Sacks DB. The calcium-sensing receptor regulates the NLRP3 inflammasome through Ca^2+^ and cAMP. Nature. (2012) 492:123–7. 10.1038/nature11588 23143333 PMC4175565

[68] Yaron JR Gangaraju S Rao MY Kong X Zhang L Su F. K(+) regulates Ca(2+) to drive inflammasome signaling: dynamic visualization of ion flux in live cells. Cell Death Dis. (2015) 6:e1954. 10.1038/cddis.2015.277 26512962 PMC5399176

[69] Tang T Lang X Xu C Wang X Gong T Yang Y. CLICs-dependent chloride efflux is an essential and proximal upstream event for NLRP3 inflammasome activation. Nat Commun. (2017) 8:202. 10.1038/s41467-017-00227-x 28779175 PMC5544706

[70] Domingo-Fernández R Coll RC Kearney J Breit S O'Neill LAJ. The intracellular chloride channel proteins CLIC1 and CLIC4 induce IL-1β transcription and activate the NLRP3 inflammasome. J Biol Chem. (2017) 292:12077–87. 10.1074/jbc.M117.797126 28576828 PMC5519359

[71] de Gassart A Martinon F. Pyroptosis: caspase-11 unlocks the gates of death. Immunity. (2015) 43:835–7. 10.1016/j.immuni.2015.10.024 26588774

[72] Bergsbaken T Fink SL Cookson BT. Pyroptosis: host cell death and inflammation. Nat Rev Microbiol. (2009) 7:99–109. 10.1038/nrmicro2070 19148178 PMC2910423

[73] McIntire CR Yeretssian G Saleh M. Inflammasomes in infection and inflammation. Apoptosis. (2009) 14:522–35. 10.1007/s10495-009-0312-3 19156527

[74] Shi J Zhao Y Wang K Shi X Wang Y Huang H. Cleavage of GSDMD by inflammatory caspases determines pyroptotic cell death. Nature. (2015) 526:660–5. 10.1038/nature15514 26375003

[75] He WT Wan H Hu L Chen P Wang X Huang Z. Gasdermin D is an executor of pyroptosis and required for interleukin-1β secretion. Cell Res. (2015) 25:1285–98. 10.1038/cr.2015.139 26611636 PMC4670995

[76] Monteleone M Stanley AC Chen KW Brown DL Bezbradica JS von Pein JB. Interleukin-1β maturation triggers its relocation to the plasma membrane for gasdermin-D-dependent and -independent secretion. Cell Rep. (2018) 24:1425–33. 10.1016/j.celrep.2018.07.027 30089254

[77] Evavold CL Ruan J Tan Y Xia S Wu H Kagan JC. The pore-forming protein gasdermin D regulates interleukin-1 secretion from living macrophages. Immunity. (2018) 48:35–44.e6. 10.1016/j.immuni.2017.11.013 29195811 PMC5773350

[78] Doitsh G Greene WC. Dissecting how CD4 T cells are lost during HIV infection. Cell Host Microbe. (2016) 19:280–91. 10.1016/j.chom.2016.02.012 26962940 PMC4835240

[79] Galloway NL Doitsh G Monroe KM Yang Z Muñoz-Arias I Levy DN. Cell-to-cell transmission of HIV-1 is required to trigger pyroptotic death of lymphoid-tissue-derived CD4 T cells. Cell Rep. (2015) 12:1555–63. 10.1016/j.celrep.2015.08.011 26321639 PMC4565731

[80] Doitsh G Galloway NL Geng X Yang Z Monroe KM Zepeda O. Cell death by pyroptosis drives CD4 T-cell depletion in HIV-1 infection. Nature. (2014) 505:509–14. 10.1038/nature12940 24356306 PMC4047036

[81] Monroe KM Yang Z Johnson JR Geng X Doitsh G Krogan NJ. IFI16 DNA sensor is required for death of lymphoid CD4 T cells abortively infected with HIV. Science. (2014) 343:428–32. 10.1126/science.1243640 24356113 PMC3976200

[82] Doitsh G Cavrois M Lassen KG Zepeda O Yang Z Santiago ML. Abortive HIV infection mediates CD4 T cell depletion and inflammation in human lymphoid tissue. Cell. (2010) 143:789–801. 10.1016/j.cell.2010.11.001 21111238 PMC3026834

[83] Zhang J Han Y Shi H Chen J Zhang X Wang X. Swine acute diarrhea syndrome coronavirus-induced apoptosis is caspase- and cyclophilin D- dependent. Emerg Microbes Infect. (2020) 9:439–56. 10.1080/22221751.2020.1722758 32090691 PMC7054944

[84] Siu KL Yuen KS Castaño-Rodriguez C Ye ZW Yeung ML Fung SY. Severe acute respiratory syndrome coronavirus ORF3a protein activates the NLRP3 inflammasome by promoting TRAF3-dependent ubiquitination of ASC. FASEB J. (2019) 33:8865–77. 10.1096/fj.201802418R 31034780 PMC6662968

[85] Ahn M Anderson DE Zhang Q Tan CW Lim BL Luko K. Dampened NLRP3-mediated inflammation in bats and implications for a special viral reservoir host. Nat Microbiol. (2019) 4:789–99. 10.1038/s41564-019-0371-3 30804542 PMC7096966

[86] Yue Y Nabar NR Shi CS Kamenyeva O Xiao X Hwang IY. SARS-coronavirus open reading frame-3a drives multimodal necrotic cell death. Cell Death Dis. (2018) 9:904. 10.1038/s41419-018-0917-y 30185776 PMC6125346

[87] DeDiego ML Nieto-Torres JL Jimenez-Guardeño JM Regla-Nava JA Castaño-Rodriguez C Fernandez-Delgado R. Coronavirus virulence genes with main focus on SARS-CoV envelope gene. Virus Res. (2014) 194:124–37. 10.1016/j.virusres.2014.07.024 25093995 PMC4261026

[88] Tan YJ Lim SG Hong W. Regulation of cell death during infection by the severe acute respiratory syndrome coronavirus and other coronaviruses. Cell Microbiol. (2007) 9:2552–61. 10.1111/j.1462-5822.2007.01034.x 17714515 PMC7162196

[89] Patton LM Saggart BS Ahmed NK Leff JA Repine JE. Interleukin-1 beta-induced neutrophil recruitment and acute lung injury in hamsters. Inflammation. (1995) 19:23–9. 10.1007/BF01534377 7705884

[90] Kolb M Margetts PJ Anthony DC Pitossi F Gauldie J. Transient expression of IL-1beta induces acute lung injury and chronic repair leading to pulmonary fibrosis. J Clin Invest. (2001) 107:1529–36. 10.1172/JCI12568 11413160 PMC200196

[91] Meduri GU Headley S Kohler G Stentz F Tolley E Umberger R. Persistent elevation of inflammatory cytokines predicts a poor outcome in ARDS. Plasma IL-1 beta and IL-6 levels are consistent and efficient predictors of outcome over time. Chest. (1995) 107:1062–73. 10.1378/chest.107.4.1062 7705118

[92] Meduri GU Kohler G Headley S Tolley E Stentz F Postlethwaite A. Inflammatory cytokines in the BAL of patients with ARDS. Persistent elevation over time predicts poor outcome. Chest. (1995) 108:1303–14. 10.1378/chest.108.5.1303 7587434

[93] Park WY Goodman RB Steinberg KP Ruzinski JT Radella F Park DR. Cytokine balance in the lungs of patients with acute respiratory distress syndrome. Am J Respir Crit Care Med. (2001) 164:1896–903. 10.1164/ajrccm.164.10.2104013 11734443

[94] Bouros D Alexandrakis MG Antoniou KM Agouridakis P Pneumatikos I Anevlavis S. The clinical significance of serum and bronchoalveolar lavage inflammatory cytokines in patients at risk for acute respiratory distress syndrome. BMC Pulm Med. (2004) 4:6. 10.1186/1471-2466-4-6 15315713 PMC516781

[95] Lau SKP Lau CCY Chan KH Li CPY Chen H Jin DY. Delayed induction of proinflammatory cytokines and suppression of innate antiviral response by the novel middle east respiratory syndrome coronavirus: implications for pathogenesis and treatment. J Gen Virol. (2013) 94:2679–90. 10.1099/vir.0.055533-0 24077366

[96] Min CK Cheon S Ha NY Sohn KM Kim Y Aigerim A. Comparative and kinetic analysis of viral shedding and immunological responses in MERS patients representing a broad spectrum of disease severity. Sci Rep. (2016) 6:25359. 10.1038/srep25359 27146253 PMC4857172

[97] Alosaimi B Hamed ME Naeem A Alsharef AA AlQahtani SY AlDosari KM. MERS-CoV infection is associated with downregulation of genes encoding Th1 and Th2 cytokines/chemokines and elevated inflammatory innate immune response in the lower respiratory tract. Cytokine. (2020) 126:154895. 10.1016/j.cyto.2019.154895 31706200 PMC7128721

[98] Beigel JH Farrar J Han AM Hayden FG Hyer R de Jong MD. Avian influenza A (H5N1) infection in humans. N Engl J Med. (2005) 353:1374–85. 10.1056/NEJMra052211 16192482

[99] Tumpey TM Basler CF Aguilar PV Zeng H Solórzano A Swayne DE. Characterization of the reconstructed 1918 Spanish influenza pandemic virus. Science. (2005) 310:77–80. 10.1126/science.1119392 16210530

[100] Perrone LA Plowden JK García-Sastre A Katz JM Tumpey TM. H5N1 and 1918 pandemic influenza virus infection results in early and excessive infiltration of macrophages and neutrophils in the lungs of mice. PLoS Pathog. (2008) 4:e1000115. 10.1371/journal.ppat.1000115 18670648 PMC2483250

[101] Kobasa D Jones SM Shinya K Kash JC Copps J Ebihara H. Aberrant innate immune response in lethal infection of macaques with the 1918 influenza virus. Nature. (2007) 445:319–23. 10.1038/nature05495 17230189

[102] Schmitz N Kurrer M Bachmann MF Kopf M. Interleukin-1 is responsible for acute lung immunopathology but increases survival of respiratory influenza virus infection. J Virol. (2005) 79:6441–8. 10.1128/JVI.79.10.6441-6448.2005 15858027 PMC1091664

[103] Gasse P Mary C Guenon I Noulin N Charron S Schnyder-Candrian S. IL-1R1/MyD88 signaling and the inflammasome are essential in pulmonary inflammation and fibrosis in mice. J Clin Invest. (2007) 117:3786–99. 10.1172/JCI32285 17992263 PMC2066195

[104] Kim KS Jung H Shin IK Choi BR Kim DH. Induction of interleukin-1 beta (IL-1β) is a critical component of lung inflammation during influenza A (H1N1) virus infection. J Med Virol. (2015) 87:1104–12. 10.1002/jmv.24138 25802122

[105] Xu H Zhong L Deng J Peng J Dan H Zeng X. High expression of ACE2 receptor of 2019-nCoV on the epithelial cells of oral mucosa. Int J Oral Sci. (2020) 12:8. 10.1038/s41368-020-0074-x 32094336 PMC7039956

[106] Fu B Xu X Wei H. Why tocilizumab could be an effective treatment for severe COVID-19? J Transl Med. (2020) 18:164. 10.1186/s12967-020-02339-3 32290839 PMC7154566

[107] Fung SY Yuen KS Ye ZW Chan CP Jin DY. A tug-of-war between severe acute respiratory syndrome coronavirus 2 and host antiviral defence: lessons from other pathogenic viruses. Emerg Microbes Infect. (2020) 9:558–70. 10.1080/22221751.2020.1736644 32172672 PMC7103735

[108] Channappanavar R Fehr AR Vijay R Mack M Zhao J Meyerholz DK. Dysregulated type I interferon and inflammatory monocyte-macrophage responses cause lethal pneumonia in SARS-CoV-infected mice. Cell Host Microbe. (2016) 19:181–93. 10.1016/j.chom.2016.01.007 26867177 PMC4752723

[109] Nieto-Torres JL DeDiego ML Verdiá-Báguena C Jimenez-Guardeño JM Regla-Nava JA Fernandez-Delgado R. Severe acute respiratory syndrome coronavirus envelope protein ion channel activity promotes virus fitness and pathogenesis. PLoS Pathog. (2014) 10:e1004077. 10.1371/journal.ppat.1004077 24788150 PMC4006877

[110] Fu Y Cheng Y Wu Y. Understanding SARS-CoV-2-mediated inflammatory responses: from mechanisms to potential therapeutic tools. Virol Sin. (2020). 10.1007/s12250-020-00207-4. [Epub ahead of print]. 32125642 PMC7090474

[111] Blanco-Melo D Nilsson-Payant BE Liu WC Uhl S Hoagland D Møller R. Imbalanced host response to SARS-CoV-2 drives development of COVID-19. Cell. (2020) 181:1036–45.e9. 10.1016/j.cell.2020.04.026 32416070 PMC7227586

[112] Smits SL de Lang A van den Brand JM Leijten LM van IJcken WF Eijkemans MJ. Exacerbated innate host response to SARS-CoV in aged non-human primates. PLoS Pathog. (2010) 6:e1000756. 10.1371/journal.ppat.1000756 20140198 PMC2816697

[113] Barker BR Taxman DJ Ting JP. Cross-regulation between the IL-1β/IL-18 processing inflammasome and other inflammatory cytokines. Curr Opin Immunol. (2011) 23:591–7. 10.1016/j.coi.2011.07.005 21839623 PMC3380339

[114] Zhang S Li L Shen A Chen Y Qi Z. Rational use of tocilizumab in the treatment of novel coronavirus pneumonia. Clin Drug Investig. (2020) 40:511–8. 10.1007/s40261-020-00917-3 32337664 PMC7183818

[115] Chen G Wu D Guo W Cao Y Huang D Wang H. Clinical and immunological features of severe and moderate coronavirus disease 2019. J Clin Invest. (2020) 130:2620–9. 10.1172/JCI137244 32217835 PMC7190990

[116] An PJ Yi ZZ Yang LP. Biochemical indicators of coronavirus disease 2019 exacerbation and the clinical implications. Pharmacol Res. (2020) 159:104946. 10.1016/j.phrs.2020.104946 32450346 PMC7244444

[117] Merad M Martin JC. Pathological inflammation in patients with COVID-19: a key role for monocytes and macrophages. Nat Rev Immunol. (2020) 20:355–62. 10.1038/s41577-020-0331-4 32376901 PMC7201395

[118] Liao M Liu Y Yuan J Wen Y Xu G Zhao J. Single-cell landscape of bronchoalveolar immune cells in patients with COVID-19. Nat Med. (2020). 10.1038/s41591-020-0901-9 32398875

[119] Franchi L Eigenbrod T Núñez G. Cutting edge: TNF-alpha mediates sensitization to ATP and silica via the NLRP3 inflammasome in the absence of microbial stimulation. J Immunol. (2009) 183:792–6. 10.4049/jimmunol.0900173 19542372 PMC2754237

[120] Zhang H Penninger JM Li Y Zhong N Slutsky AS. Angiotensin-converting enzyme 2 (ACE2) as a SARS-CoV-2 receptor: molecular mechanisms and potential therapeutic target. Intensive Care Med. (2020) 46:586–90. 10.1007/s00134-020-05985-9 32125455 PMC7079879

[121] Verdecchia P Cavallini C Spanevello A Angeli F. The pivotal link between ACE2 deficiency and SARS-CoV-2 infection. Eur J Intern Med. (2020) 76:14–20. 10.1016/j.ejim.2020.04.037 32336612 PMC7167588

[122] Glowacka I Bertram S Herzog P Pfefferle S Steffen I Muench MO. Differential downregulation of ACE2 by the spike proteins of severe acute respiratory syndrome coronavirus and human coronavirus NL63. J Virol. (2010) 84:1198–205. 10.1128/JVI.01248-09 19864379 PMC2798380

[123] Nieto-Torres JL Verdiá-Báguena C Jimenez-Guardeño JM Regla-Nava JA Castaño-Rodriguez C Fernandez-Delgado R. Severe acute respiratory syndrome coronavirus E protein transports calcium ions and activates the NLRP3 inflammasome. Virology. (2015) 485:330–9. 10.1016/j.virol.2015.08.010 26331680 PMC4619128

[124] Lu W Zheng BJ Xu K Schwarz W Du L Wong CK. Severe acute respiratory syndrome-associated coronavirus 3a protein forms an ion channel and modulates virus release. Proc Natl Acad Sci USA. (2006) 103:12540–5. 10.1073/pnas.0605402103 16894145 PMC1567914

[125] Chen CC Krüger J Sramala I Hsu HJ Henklein P Chen YM. ORF8a of SARS-CoV forms an ion channel: experiments and molecular dynamics simulations. Biochim Biophys Acta. (2011) 1808:572–9. 10.1016/j.bbamem.2010.08.004 20708597 PMC7094593

[126] Castaño-Rodriguez C Honrubia JM Gutiérrez-Álvarez J DeDiego ML Nieto-Torres JL Jimenez-Guardeño JM. Role of severe acute respiratory syndrome coronavirus viroporins E, 3a, and 8a in replication and pathogenesis. mBio. (2018) 9:e02325-17. 10.1128/mBio.02325-17 29789363 PMC5964350

[127] Shi CS Nabar NR Huang NN Kehrl JH. SARS-coronavirus open reading frame-8b triggers intracellular stress pathways and activates NLRP3 inflammasomes. Cell Death Discov. (2019) 5:101. 10.1038/s41420-019-0181-7 31231549 PMC6549181

[128] Astuti I Ysrafil. Severe acute respiratory syndrome coronavirus 2 (SARS-CoV-2): an overview of viral structure and host response. Diabetes Metab Syndr. (2020) 14:407–12. 10.1016/j.dsx.2020.04.020 32335367 PMC7165108

[129] Velazquez-Salinas L Zarate S Eberl S Gladue DP Novella I Borca MV Positive selection of ORF3a and ORF8 genes drives the evolution of SARS-CoV-2 during the 2020 COVID-19 pandemic. bioRxiv [preprint]. (2020). 10.1101/2020.04.10.035964 PMC7644918 33193132

[130] Lau SK Feng Y Chen H Luk HK Yang WH Li KS. Severe acute respiratory syndrome (SARS) Coronavirus ORF8 protein is acquired from SARS-related coronavirus from greater horseshoe bats through recombination. J Virol. (2015) 89:10532–47. 10.1128/JVI.01048-15 26269185 PMC4580176

[131] Pachetti M Marini B Benedetti F Giudici F Mauro E Storici P. Emerging SARS-CoV-2 mutation hot spots include a novel RNA-dependent-RNA polymerase variant. J Transl Med. (2020) 18:179. 10.1186/s12967-020-02344-6 32321524 PMC7174922

[132] Segovia M Russo S Jeldres M Mahmoud YD Perez V Duhalde M. Targeting TMEM176B enhances antitumor immunity and augments the efficacy of immune checkpoint blockers by unleashing inflammasome activation. Cancer Cell. (2019) 35:767–81.e6. 10.1016/j.ccell.2019.04.003 31085177 PMC6521897

[133] Segovia M Russo S Girotti MR Rabinovich GA Hill M. Role of inflammasome activation in tumor immunity triggered by immune checkpoint blockers. Clin Exp Immunol. (2020) 200:155–62. 10.1111/cei.13433 32297328 PMC7160664

[134] Mullard A. NLRP3 inhibitors stoke anti-inflammatory ambitions. Nat Rev Drug Discov. (2019) 18:405–7. 10.1038/d41573-019-00086-9 31160775

[135] Yang Y Wang H Kouadir M Song H Shi F. Recent advances in the mechanisms of NLRP3 inflammasome activation and its inhibitors. Cell Death Dis. (2019) 10:128. 10.1038/s41419-019-1413-8 30755589 PMC6372664

[136] Mangan MSJ Olhava EJ Roush WR Seidel HM Glick GD Latz E Targeting the NLRP3 inflammasome in inflammatory diseases. Nat Rev Drug Discov. (2018) 17:688 10.1038/nrd.2018.97 30116046

[137] Xu L Zhang C Jiang N He D Bai Y Xin Y. Rapamycin combined with MCC950 to treat multiple sclerosis in experimental autoimmune encephalomyelitis. J Cell Biochem. (2019) 120:5160–8. 10.1002/jcb.27792 30320900

[138] Perregaux DG McNiff P Laliberte R Hawryluk N Peurano H Stam E. Identification and characterization of a novel class of interleukin-1 post-translational processing inhibitors. J Pharmacol Exp Ther. (2001) 299:187–97. 11561079

[139] Gordon R Albornoz EA Christie DC Langley MR Kumar V Mantovani S. Inflammasome inhibition prevents α-synuclein pathology and dopaminergic neurodegeneration in mice. Sci Transl Med. (2018) 10:eaah4066. 10.1126/scitranslmed.aah4066 30381407 PMC6483075

[140] Toldo S Mauro AG Cutter Z Van Tassell BW Mezzaroma E Del Buono MG. The NLRP3 inflammasome inhibitor, OLT1177 (Dapansutrile), reduces infarct size and preserves contractile function after ischemia reperfusion injury in the mouse. J Cardiovasc Pharmacol. (2019) 73:215–22. 10.1097/FJC.0000000000000658 30747785

[141] Sánchez-Fernández A Skouras DB Dinarello CA López-Vales R. OLT1177 (Dapansutrile), a selective NLRP3 inflammasome inhibitor, ameliorates experimental autoimmune encephalomyelitis pathogenesis. Front Immunol. (2019) 10:2578. 10.3389/fimmu.2019.02578 31736980 PMC6839275

[142] Marchetti C Swartzwelter B Gamboni F Neff CP Richter K Azam T. OLT1177, a β-sulfonyl nitrile compound, safe in humans, inhibits the NLRP3 inflammasome and reverses the metabolic cost of inflammation. Proc Natl Acad Sci USA. (2018) 115:E1530–9. 10.1073/pnas.1716095115 29378952 PMC5816172

[143] Marchetti C Swartzwelter B Koenders MI Azam T Tengesdal IW Powers N. NLRP3 inflammasome inhibitor OLT1177 suppresses joint inflammation in murine models of acute arthritis. Arthritis Res Ther. (2018) 20:169. 10.1186/s13075-018-1664-2 30075804 PMC6091035

[144] Ridker PM Everett BM Thuren T MacFadyen JG Chang WH Ballantyne C. Antiinflammatory therapy with canakinumab for atherosclerotic disease. N Engl J Med. (2017) 377:1119–31. 10.1056/NEJMoa1707914 28845751

[145] Zheng ZH Zeng X Nie XY Cheng YJ Liu J Lin XX. Interleukin-1 blockade treatment decreasing cardiovascular risk. Clin Cardiol. (2019) 42:942–51. 10.1002/clc.23246 31415103 PMC6788469

[146] Ikonomidis I Tzortzis S Lekakis J Paraskevaidis I Andreadou I Nikolaou M. Lowering interleukin-1 activity with anakinra improves myocardial deformation in rheumatoid arthritis. Heart. (2009) 95:1502–7. 10.1136/hrt.2009.168971 19482847

[147] Gómez-García F Sanz-Cabanillas JL Viguera-Guerra I Isla-Tejera B Nieto AV Ruano J. Scoping review on use of drugs targeting interleukin 1 pathway in DIRA and DITRA. Dermatol Ther. (2018) 8:539–56. 10.1007/s13555-018-0269-7 30392030 PMC6261121

[148] Schumacher HR Sundy JS Terkeltaub R Knapp HR Mellis SJ Stahl N. Rilonacept (interleukin-1 trap) in the prevention of acute gout flares during initiation of urate-lowering therapy: results of a phase II randomized, double-blind, placebo-controlled trial. Arthritis Rheum. (2012) 64:876–84. 10.1002/art.33412 22223180

[149] Kapur S Bonk ME. Rilonacept (arcalyst), an interleukin-1 trap for the treatment of cryopyrin-associated periodic syndromes. P T. (2009) 34:138–41. 19561849 PMC2697082

[150] Knickelbein JE Tucker WR Bhatt N Armbrust K Valent D Obiyor D. Gevokizumab in the treatment of autoimmune non-necrotizing anterior scleritis: results of a phase I/II clinical trial. Am J Ophthalmol. (2016) 172:104–10. 10.1016/j.ajo.2016.09.017 27663070 PMC5121021

[151] Owyang AM Issafras H Corbin J Ahluwalia K Larsen P Pongo E. XOMA 052, a potent, high-affinity monoclonal antibody for the treatment of IL-1β-mediated diseases. MAbs. (2011) 3:49–60. 10.4161/mabs.3.1.13989 21048425 PMC3038011

[152] Blech M Peter D Fischer P Bauer MM Hafner M Zeeb M. One target-two different binding modes: structural insights into gevokizumab and canakinumab interactions to interleukin-1β. J Mol Biol. (2013) 425:94–111. 10.1016/j.jmb.2012.09.021 23041424

[153] Murthy H Iqbal M Chavez JC Kharfan-Dabaja MA. Cytokine release syndrome: current perspectives. Immunotargets Ther. (2019) 8:43–52. 10.2147/ITT.S202015 31754614 PMC6825470

[154] Shimabukuro-Vornhagen A Gödel P Subklewe M Stemmler HJ Schlößer HA Schlaak M. Cytokine release syndrome. J Immunother Cancer. (2018) 6:56. 10.1186/s40425-018-0343-9 29907163 PMC6003181

[155] Chatenoud L Ferran C Reuter A Legendre C Gevaert Y Kreis H. Systemic reaction to the anti-T-cell monoclonal antibody OKT3 in relation to serum levels of tumor necrosis factor and interferon-gamma [corrected]. N Engl J Med. (1989) 320:1420–1. 10.1056/NEJM198905253202117 2785642

[156] Chatenoud L Ferran C Legendre C Thouard I Merite S Reuter A. *In vivo* cell activation following OKT3 administration. Systemic cytokine release and modulation by corticosteroids. Transplantation. (1990) 49:697–702. 10.1097/00007890-199004000-00009 2109379

[157] Pihusch R Holler E Mühlbayer D Göhring P Stötzer O Pihusch M. The impact of antithymocyte globulin on short-term toxicity after allogeneic stem cell transplantation. Bone Marrow Transplant. (2002) 30:347–54. 10.1038/sj.bmt.1703640 12235518

[158] Suntharalingam G Perry MR Ward S Brett SJ Castello-Cortes A Brunner MD. Cytokine storm in a phase 1 trial of the anti-CD28 monoclonal antibody TGN1412. N Engl J Med. (2006) 355:1018–28. 10.1056/NEJMoa063842 16908486

[159] Winkler U Jensen M Manzke O Schulz H Diehl V Engert A. Cytokine-release syndrome in patients with B-cell chronic lymphocytic leukemia and high lymphocyte counts after treatment with an anti-CD20 monoclonal antibody (rituximab, IDEC-C2B8). Blood. (1999) 94:2217–24. 10.1182/blood.V94.7.2217.419k02_2217_2224 10498591

[160] Freeman CL Morschhauser F Sehn L Dixon M Houghton R Lamy T. Cytokine release in patients with CLL treated with obinutuzumab and possible relationship with infusion-related reactions. Blood. (2015) 126:2646–9. 10.1182/blood-2015-09-670802 26447188 PMC4671111

[161] Wing MG Moreau T Greenwood J Smith RM Hale G Isaacs J. Mechanism of first-dose cytokine-release syndrome by CAMPATH 1-H: involvement of CD16 (FcgammaRIII) and CD11a/CD18 (LFA-1) on NK cells. J Clin Invest. (1996) 98:2819–26. 10.1172/JCI119110 8981930 PMC507749

[162] Alig SK Dreyling M Seppi B Aulinger B Witkowski L Rieger CT. Severe cytokine release syndrome after the first dose of Brentuximab Vedotin in a patient with relapsed systemic anaplastic large cell lymphoma (sALCL): a case report and review of literature. Eur J Haematol. (2015) 94:554–7. 10.1111/ejh.12396 24913471

[163] de Vos S Forero-Torres A Ansell SM Kahl B Cheson BD Bartlett NL. A phase II study of dacetuzumab (SGN-40) in patients with relapsed diffuse large B-cell lymphoma (DLBCL) and correlative analyses of patient-specific factors. J Hematol Oncol. (2014) 7:44. 10.1186/1756-8722-7-44 24919462 PMC4065310

[164] Rotz SJ Leino D Szabo S Mangino JL Turpin BK Pressey JG. Severe cytokine release syndrome in a patient receiving PD-1-directed therapy. Pediatr Blood Cancer. (2017) 64. 10.1002/pbc.26642 28544595

[165] Tonini G Santini D Vincenzi B Borzomati D Dicuonzo G La Cesa A. Oxaliplatin may induce cytokine-release syndrome in colorectal cancer patients. J Biol Regul Homeost Agents. (2002) 16:105–9. 12144121

[166] Aue G Njuguna N Tian X Soto S Hughes T Vire B. Lenalidomide-induced upregulation of CD80 on tumor cells correlates with T-cell activation, the rapid onset of a cytokine release syndrome and leukemic cell clearance in chronic lymphocytic leukemia. Haematologica. (2009) 94:1266–73. 10.3324/haematol.2009.005835 19734418 PMC2738719

[167] Abboud R Keller J Slade M DiPersio JF Westervelt P Rettig MP. Severe cytokine-release syndrome after T cell-replete peripheral blood haploidentical donor transplantation is associated with poor survival and anti-IL-6 therapy is safe and well tolerated. Biol Blood Marrow Transplant. (2016) 22:1851–60. 10.1016/j.bbmt.2016.06.010 27318038 PMC5070661

[168] Cho C Perales MA. Rapid identification of cytokine release syndrome after haploidentical PBSC transplantation and successful therapy with tocilizumab. Bone Marrow Transplant. (2016) 51:1620–1. 10.1038/bmt.2016.229 27668764 PMC5924416

[169] Lundemose JB Smith H Sweet C. Cytokine release from human peripheral blood leucocytes incubated with endotoxin with and without prior infection with influenza virus: relevance to the sudden infant death syndrome. Int J Exp Pathol. (1993) 74:291–7. 8392861 PMC2002163

[170] Porter D Frey N Wood PA Weng Y Grupp SA. Grading of cytokine release syndrome associated with the CAR T cell therapy tisagenlecleucel. J Hematol Oncol. (2018) 11:35. 10.1186/s13045-018-0571-y 29499750 PMC5833070

[171] Wu Z McGoogan JM. Characteristics of and important lessons from the coronavirus disease 2019 (COVID-19) outbreak in china: summary of a report of 72 314 cases from the chinese center for disease control and prevention. JAMA. (2020) 323:1239–42. 10.1001/jama.2020.2648 32091533

[172] Ruan Q Yang K Wang W Jiang L Song J. Clinical predictors of mortality due to COVID-19 based on an analysis of data of 150 patients from Wuhan, China. Intensive Care Med. (2020) 46:846–8. 10.1007/s00134-020-05991-x 32125452 PMC7080116

[173] Qin C Zhou L Hu Z Zhang S Yang S Tao Y. Dysregulation of immune response in patients with COVID-19 in Wuhan, China. Clin Infect Dis. (2020). 10.2139/ssrn.3541136. [Epub ahead of print]. 32161940 PMC7108125

[174] Connors JM Levy JH. COVID-19 and its implications for thrombosis and anticoagulation. Blood. (2020) 135:2033–40. 10.1182/blood.2020006000 32339221 PMC7273827

[175] Helms J Tacquard C Severac F Leonard-Lorant I Ohana M Delabranche X. High risk of thrombosis in patients with severe SARS-CoV-2 infection: a multicenter prospective cohort study. Intensive Care Med. (2020) 46:1089–98. 10.1007/s00134-020-06062-x 32367170 PMC7197634

[176] Zhang Y Xiao M Zhang S Xia P Cao W Jiang W. Coagulopathy and antiphospholipid antibodies in patients with Covid-19. N Engl J Med. (2020) 382:e38. 10.1056/NEJMc2007575 32268022 PMC7161262

[177] Wu C Lu W Zhang Y Zhang G Shi X Hisada Y. Inflammasome activation triggers blood clotting and host death through pyroptosis. Immunity. (2019) 50:1401–1411.e4. 10.1016/j.immuni.2019.04.003 31076358 PMC6791531

[178] Bortolotti P Faure E Kipnis E. Inflammasomes in tissue damages and immune disorders after trauma. Front Immunol. (2018) 9:1900. 10.3389/fimmu.2018.01900 30166988 PMC6105702

[179] Qiao J Wu X Luo Q Wei G Xu M Wu Y. NLRP3 regulates platelet integrin αIIbβ3 outside-in signaling, hemostasis and arterial thrombosis. Haematologica. (2018) 103:1568–76. 10.3324/haematol.2018.191700 29794149 PMC6119128

[180] Murthy P Durco F Miller-Ocuin JL Takedai T Shankar S Liang X. The NLRP3 inflammasome and bruton's tyrosine kinase in platelets co-regulate platelet activation, aggregation, and in vitro thrombus formation. Biochem Biophys Res Commun. (2017) 483:230–6. 10.1016/j.bbrc.2016.12.161 28034752

[181] Gupta N Sahu A Prabhakar A Chatterjee T Tyagi T Kumari B. Activation of NLRP3 inflammasome complex potentiates venous thrombosis in response to hypoxia. Proc Natl Acad Sci USA. (2017) 114:4763–8. 10.1073/pnas.1620458114 28420787 PMC5422823

[182] DeDiego ML Nieto-Torres JL Regla-Nava JA Jimenez-Guardeño JM Fernandez-Delgado R Fett C. Inhibition of NF-κB-mediated inflammation in severe acute respiratory syndrome coronavirus-infected mice increases survival. J Virol. (2014) 88:913–24. 10.1128/JVI.02576-13 24198408 PMC3911641

[183] Ward SE Loutfy MR Blatt LM Siminovitch KA Chen J Hinek A. Dynamic changes in clinical features and cytokine/chemokine responses in SARS patients treated with interferon alfacon-1 plus corticosteroids. Antivir Ther. (2005) 10:263–75. 15865221

[184] Loutfy MR Blatt LM Siminovitch KA Ward S Wolff B Lho H. Interferon alfacon-1 plus corticosteroids in severe acute respiratory syndrome: a preliminary study. JAMA. (2003) 290:3222–8. 10.1001/jama.290.24.3222 14693875

[185] Shakoory B Carcillo JA Chatham WW Amdur RL Zhao H Dinarello CA. Interleukin-1 receptor blockade is associated with reduced mortality in sepsis patients with features of macrophage activation syndrome: reanalysis of a prior phase III trial. Crit Care Med. (2016) 44:275–81. 10.1097/CCM.0000000000001402 26584195 PMC5378312

[186] Norelli M Camisa B Barbiera G Falcone L Purevdorj A Genua M. Monocyte-derived IL-1 and IL-6 are differentially required for cytokine-release syndrome and neurotoxicity due to CAR T cells. Nat Med. (2018) 24:739–48. 10.1038/s41591-018-0036-4 29808007

[187] Le RQ Li L Yuan W Shord SS Nie L Habtemariam BA. FDA approval summary: tocilizumab for treatment of chimeric antigen receptor T cell-induced severe or life-threatening cytokine release syndrome. Oncologist. (2018) 23:943–7. 10.1634/theoncologist.2018-0028 29622697 PMC6156173

[188] Kotch C Barrett D Teachey DT. Tocilizumab for the treatment of chimeric antigen receptor T cell-induced cytokine release syndrome. Expert Rev Clin Immunol. (2019) 15:813–22. 10.1080/1744666X.2019.1629904 31219357 PMC7936577

[189] Giavridis T van der Stegen SJC Eyquem J Hamieh M Piersigilli A Sadelain M. CAR T cell-induced cytokine release syndrome is mediated by macrophages and abated by IL-1 blockade. Nat Med. (2018) 24:731–8. 10.1038/s41591-018-0041-7 29808005 PMC6410714

[190] Vijay R Fehr AR Janowski AM Athmer J Wheeler DL Grunewald M. Virus-induced inflammasome activation is suppressed by prostaglandin D. Proc Natl Acad Sci USA. (2017) 114:E5444–E5453. 10.1073/pnas.1704099114 28630327 PMC5502630

[191] Cavalli G Luca GD Campochiaro C Della-Torre E Ripa M Canetti D. Interleukin-1 blockade with high-dose anakinra in patients with COVID-19, acute respiratory distress syndrome, and hyperinflammation: a retrospective cohort study. Lancet Rheumatol. (2020) 2:e310-1. 10.1016/S2665-9913.(20)30127-2 32501454 PMC7252085

[192] Abderrazak A Couchie D Mahmood DF Elhage R Vindis C Laffargue M. Anti-inflammatory and antiatherogenic effects of the NLRP3 inflammasome inhibitor arglabin in ApoE2.Ki mice fed a high-fat diet. Circulation. (2015) 131:1061–70. 10.1161/CIRCULATIONAHA.114.013730 25613820

[193] Abbate A Van Tassell BW Biondi-Zoccai G Kontos MC Grizzard JD Spillman DW. Effects of interleukin-1 blockade with anakinra on adverse cardiac remodeling and heart failure after acute myocardial infarction [from the Virginia Commonwealth University-Anakinra Remodeling Trial (2) (VCU-ART2) pilot study]. Am J Cardiol. (2013) 111:1394–400. 10.1016/j.amjcard.2013.01.287 23453459 PMC3644511

[194] Abbate A Van Tassell BW Seropian IM Toldo S Robati R Varma A. Interleukin-1beta modulation using a genetically engineered antibody prevents adverse cardiac remodelling following acute myocardial infarction in the mouse. Eur J Heart Fail. (2010) 12:319–22. 10.1093/eurjhf/hfq017 20335350

[195] Liu D Zeng X Li X Mehta JL Wang X. Role of NLRP3 inflammasome in the pathogenesis of cardiovascular diseases. Basic Res Cardiol. (2017) 113:5. 10.1007/s00395-017-0663-9 29224086

[196] Zhao M. Cytokine storm and immunomodulatory therapy in COVID-19: role of chloroquine and anti-IL-6 monoclonal antibodies. Int J Antimicrob Agents. (2020) 55:105982. 10.1016/j.ijantimicag.2020.105982 32305588 PMC7161506

[197] Liu J Cao R Xu M Wang X Zhang H Hu H. Hydroxychloroquine, a less toxic derivative of chloroquine, is effective in inhibiting SARS-CoV-2 infection *in vitro*. Cell Discov. (2020) 6:16. 10.1038/s41421-020-0156-0 32194981 PMC7078228

[198] Wang M Cao R Zhang L Yang X Liu J Xu M. Remdesivir and chloroquine effectively inhibit the recently emerged novel coronavirus (2019-nCoV) *in vitro*. Cell Res. (2020) 30:269–71. 10.1038/s41422-020-0282-0 32020029 PMC7054408

[199] Gautret P Lagier JC Parola P Hoang VT Meddeb L Mailhe M Hydroxychloroquine and azithromycin as a treatment of COVID-19: results of an open-label non-randomized clinical trial. Int J Antimicrob Agents. (2020) 2020:105949 10.1016/j.ijantimicag.2020.105949 PMC7102549 32205204

[200] Geleris J Sun Y Platt J Zucker J Baldwin M Hripcsak G. Observational study of hydroxychloroquine in hospitalized patients with Covid-19. N Engl J Med. (2020). 10.1056/NEJMoa2012410. [Epub ahead of print]. 32379955 PMC7224609

[201] Taccone FS Gorham J Vincent JL. Hydroxychloroquine in the management of critically ill patients with COVID-19: the need for an evidence base. Lancet Respir Med. (2020) 8: P539–41. 10.1016/S2213-2600.(20)30172-7 32304640 PMC7159849

